# Aneugenic Effects of Epirubicin in Somatic and Germinal Cells of Male Mice

**DOI:** 10.1371/journal.pone.0109942

**Published:** 2014-10-10

**Authors:** Sabry Mohamed Attia, Sheikh Fayaz Ahmad, Radwa Mohamed Okash, Saleh Abdulrahman Bakheet

**Affiliations:** 1 Department of Pharmacology and Toxicology, College of Pharmacy, King Saud University, Riyadh, Saudi Arabia; 2 Laboratory of Chemical and Clinical Pathology, Ministry of Health, Nasr City, Cairo, Egypt; 3 Department of Pharmacology and Toxicology, College of Pharmacy, Al-Azhar University, Cairo, Egypt; University of Science and Technology of China, China

## Abstract

The ability of the antineoplastic agent epirubicin to induce aneuploidy and meiotic delay in the somatic and germinal cells of male mice was investigated by fluorescence *in situ* hybridization assay using labeled DNA probes and BrdU-incorporation assay. Mitomycin C and colchicine were used as positive controls for clastogen and aneugen, respectively, and these compounds produced the expected responses. The fluorescence *in situ* hybridization assay with a centromeric DNA probe for erythrocyte micronuclei showed that epirubicin is not only clastogenic but also aneugenic in somatic cells *in vivo*. By using the BrdU-incorporation assay, it could be shown that the meiotic delay caused by epirubicin in germ cells was approximately 48 h. Disomic and diploid sperm were shown in epididymal sperm hybridized with DNA probes specific for chromosomes 8, X and Y after epirubicin treatment. The observation that XX- and YY-sperm significantly prevailed over XY-sperm indicates missegregation during the second meiotic division. The results also suggest that earlier prophase stages contribute less to epirubicin-induced aneuploidy. Both the clastogenic and aneugenic potential of epirubicin can give rise to the development of secondary tumors and abnormal reproductive outcomes in cured cancer patients and medical personnel exposed to epirubicin.

## Introduction

In cancer therapy, surgery, radiotherapy, and chemotherapy are used frequently. Of the chemotherapeutics in clinical use, anthracyclines have a wide spectrum of antitumor activity and are clearly the most useful cancer drugs that are natural products [1]. Members of this drug group are highly active against epithelial tumors, such as carcinomas of the breast, lung, thyroid, and stomach, and also against mesenchymal malignancies such as Hodgkin's and non-Hodgkin's lymphoma, multiple myeloma, acute lymphocytic and myelogenous leukemia, and sarcomas. Anthracyclines includes many agents, such as doxorubicin. One of the developed analogues of doxorubicin is epirubicin. Epirubicin (4′-epi-doxorubicin) differs from doxorubicin in the steric position of the 4′-OH group. This drug is commonly used because it has an equivalent spectrum of antitumor action to that of doxorubicin but exhibits less systemic and cardiac toxicity [2].

Anthracyclines stabilize topoisomerase II-DNA complexes, preventing rapid turnover of the protein cross-linked DNA strand breaks, and it interferes with processes that require changes in DNA topology, such as DNA replication, repair and transcription. Other mechanisms of these agents include DNA intercalation, nuclear helicase inhibition and free radical formation [3], [4]. As a consequence of these effects, single- and double-strand breaks are introduced into the DNA. DNA damage can have a variety of biological ramifications, including inhibition of transcription and/or replication, ultimately leading to cell death [5]. As with many other anticancer drugs, high doses of anthracyclines such as doxorubicin induce apoptosis and disrupt the inner mitochondrial membrane potential [6]. However, several lines of evidence indicate that low doses of various chemotherapeutic drugs are capable of inducing mitotic catastrophe resulting from abnormal mitotic events that produce improper chromosomal segregation and cell division, leading to the formation of mutant cells [7]. These cells may develop resistance to the chemotherapeutic agents or may lead to the development of secondary tumors and abnormal reproductive outcomes [8].

Chromosomal imbalance (aneuploidy) is the most common cause of fertility impairment and fetal loss in humans. More than 35% of spontaneous abortions are due to aneuploidy, the most frequent cases being those with XO constitution (Turner syndrome) or trisomies of chromosomes 16, 21 and 22 [9]. In newborns, aneuploidy is a frequent cause of mental retardation and congenital malformations, as in the case of trisomy 21. The syndromes of missing or additional sex chromosomes carry less severe morphological defects but show drastically impaired fertility [10]. Approximately 50% of all Klinefelter syndromes (47, XXY), 80% of all Turner syndromes and 100% of cases with two Y chromosomes (47, XYY) are of paternal origin [11], while the majority of autosomal trisomies are of maternal origin [12]. Despite the increasing use of epirubicin in malignancies, no data are available in the literature on its potential aneugenicity *in vivo*. *In vitro*, epirubicin was positive for mutagenicity in the Ames test (in the presence or absence of metabolic activation) and in the HGPRT assay in V79 Chinese hamster lung fibroblasts in the absence, but not in the presence, of metabolic activation [13.14]. Similarly, *in vitro*, epirubicin was clastogenic, producing chromosome aberrations in human lymphocytes, both in the presence and absence of metabolic activation. Epirubicin was also clastogenic *in vivo*, producing structural chromosomal aberrations in a mouse bone marrow assay [15].

Epirubicin-induced gene mutation and structural chromosomal aberrations have been reported, as mentioned above, whereas epirubicin-induced numerical chromosomal aberrations (aneuploidy) have not yet been reported. Therefore, the current study was designed to delineate the potential aneuploidogenic effects of epirubicin in the somatic and germinal cells of male mice *in vivo*. Such events may have important consequences in cancer chemotherapy. First, aberrations induced in somatic cells may lead to the development of secondary tumors from cells that were not originally neoplastic. Second, aberrations induced in germ cells may be transmitted to the progeny and pose a genetic hazard to future generations. The bone marrow micronucleus test complemented by fluorescence *in situ* hybridization (FISH) assay was performed in mouse bone marrow cells to determine the aneugenic and clastogenic origin of induced micronuclei (MN). The BrdU-incorporation assay was used to test if epirubicin treatment altered the duration of the meiotic divisions, and the sperm-FISH assay was performed for aneugenicity induction during male meiosis. To determine the reliability of the methods, two-model mutagens, colchicine and mitomycin C, known to be predominantly aneugenic and clastogenic, respectively, were used as positive control substances.

## Materials and Methods

### Animals

Adult male white Swiss albino mice weighing 25–30 g (10–12 weeks old) were obtained from the Experimental Animal Care Center at our university. The animals were maintained in an air-conditioned animal house at a temperature of 25–28°C, relative humidity of ∼50% and photo-cycle of 12:12 h light and dark periods. The animals were provided with standard diet pellets and water *ad libitum*. The conduct of experiments and the procedure of sacrifice (under light ether anesthesia) were approved by the Ethics Committee of the Experimental Animal Care Society, College of Pharmacy, King Saud University, Riyadh, Saudi Arabia. Each treatment group and vehicle control group consisted of 5 randomly assigned animals.

### Drugs and treatment

Epirubicin hydrochloride (Santa Cruz Biotechnology, CA, USA) was dissolved in sterile dH_2_O. Colchicine and mitomycin C (Sigma Chemical St. Louis, MO) were dissolved in sterile dH_2_O and were used as positive controls, aneugen and clastogen, respectively. Control mice receiving sterile dH_2_O were included to code the slides and avoid scoring biases. The injected volume was 0.1 ml/10 g body weight. All other chemicals were of the finest analytical grade.

#### Bone marrow conventional micronucleus test

Animals were treated with 3, 6 or 12 mg/kg epirubicin, and bone marrow was sampled 24 h after treatment. Animal sacrifice was done under light ether anesthesia, and all efforts were made to minimize suffering. Immediately after the animal was sacrificed by cervical dislocation, both femurs were removed and bone marrow cells were sampled as described previously [16]. In human chemotherapy, epirubicin is typically administered at doses up to 135 mg/m^2^ as a single agent every three to four weeks. Mice have a body weight/surface area ratio of ≈ 3 kg/m^2^. Thus, the highest dose of 12 mg/kg used in our study corresponds to ≈ 36 mg/m^2^ and is within the dose range used for human chemotherapy. Colchicine and mitomycin C were used as the positive controls aneugen and clastogen, respectively, at a dose of 2 mg/kg each [16]. Bone marrow smears were prepared and stained with May-Gruenwald-Giemsa, as described previously [17]. At least four slides were made for each animal and allowed to dry overnight. One slide per animal was stained with May-Gruenwald/Giemsa solutions for conventional assessment of MN frequencies in polychromatic erythrocytes (PCE) and normochromatic erythrocytes (NCE). The remaining unstained slides were stored at −20°C to distinguish between clastogenic and aneugenic effects by identifying the origin of MN with mouse DNA probes. For each animal, 2,000 PCE of coded slides were scored for the presence of MN. In addition, the number of PCEs among 1000 NCE per animal was recorded to evaluate bone marrow suppression. Mitotic activity was calculated as %PCE  =  [PCE/(PCE + NCE)] x 100.

#### Bone marrow FISH analysis of MN using centromeric DNA-probe

The mouse centromeric DNA probe p^MKB6^, a 273-bp fragment that represents approximately two tandem repeats, was used in plasmid p^TZ19U^. The preparation of mouse digoxigenin-labeled DNA probe, hybridization, washing and amplification of the signals are described elsewhere [18]–[20]. The bone marrow cells were counterstained with propidium iodide (2 mg/ml) for 20 min at room temperature and cover slipped in Vectashield mounting medium. The slides were scored immediately or after storage for several days at 4°C in the dark. The presence of centromeric signals in the MN was analyzed under a fluorescence microscope equipped with an appropriate filter. Sixty-seven to 128 MN per group of coded slides were examined for the presence or absence of a centromeric DNA probe, as described previously [16].

#### Sperm BrdU-incorporation assay

To assess whether epirubicin treatment altered the timing of meiotic progression, the cells were labelled with BrdU during the last round of DNA replication before meiosis, and the presence of BrdU-labelled sperm was assessed. Mice were injected with 100 mg/kg BrdU to label spermatocytes at the S-phase during preleptotene of meiosis. During meiosis I and II, 13 days later, the mice were treated with 12 mg/kg epirubicin. Five treated and 5 solvent control mice were sacrificed per day 33 to 37 days after BrdU-injection (20–24 days after drugs treatment). Sperm were sampled from the *caudae epididymes*, and smears were prepared as described previously [21]. Decondensation of sperm heads was performed by incubation of the slides in 10 mM dithiothreitol for 30 min on ice followed by incubation in 4 mM lithium 3,5-diiodosalicylic acid for 60 min at room temperature. Immunofluorescence staining with anti-BrdU antibody and washing conditions were as described by Schmid et al. [22]. Propidium iodide (2.0 µg/ml) was used as counterstaining. Averages of 10,000 BrdU-positive and BrdU-negative cells per animal were blind-scored under a fluorescence microscope.

#### Sperm multicolor FISH assay

Random groups of five mice each were treated with single doses of 3, 6 and 12 mg/kg epirubicin. Colchicine was used as a positive control aneugen at the dose of 3 mg/kg [21]. After drug administration, the animals were maintained with food and water ad libitum until being sacrificed. Mice were sacrificed by cervical dislocation 23 days after drug treatment. To determine whether subacute treatment had an effect, because the prophase stages would be included in epirubicin treatment, doses of 0.25, 0.5 and 1 mg/kg of epirubicin were injected on 12 consecutive days and sperm was sampled 23 days after the last treatment. Immediately after animal sacrificing, the sperm were collected from the *Caudae epididymes*. The time of sampling was chosen on the basis of the BrdU incorporation study. The frequencies of disomic and diploid sperm were determined by FISH with DNA probes specific for mouse chromosomes 8, X and Y, each labelled with a different color. Hybridization, washing, and amplification of the signals were performed as described previously [21]. DAPI (4′,6-Diamidin-2′-phenylindol-dihydrochloride, 0.05–0.1 µg/ml) was used as a counterstain. Slides were examined for aneuploid sperm under a fluorescence microscope. The fluorescent signals of the coded slides were counted in multiples of ∼10,000 sperm per animal. Sperm were designated normal (X8 and Y8), disomic (X88, Y88 and XY8) or diploid (XY88, XX88 and YY88).

### Statistical analysis

Data were expressed as the mean ± standard deviation (SD) of the mean. The data were analyzed by the non-parametric tests, Kruskal-Wallis test followed by Dunn's multiple comparisons test and Mann-Whitney *U*-test using software computer program (GraphPad InStat; DATASET1.ISD). The results were considered significantly different if *P*<0.05.

## Results

### Bone marrow conventional MN test

**Figure 1:**
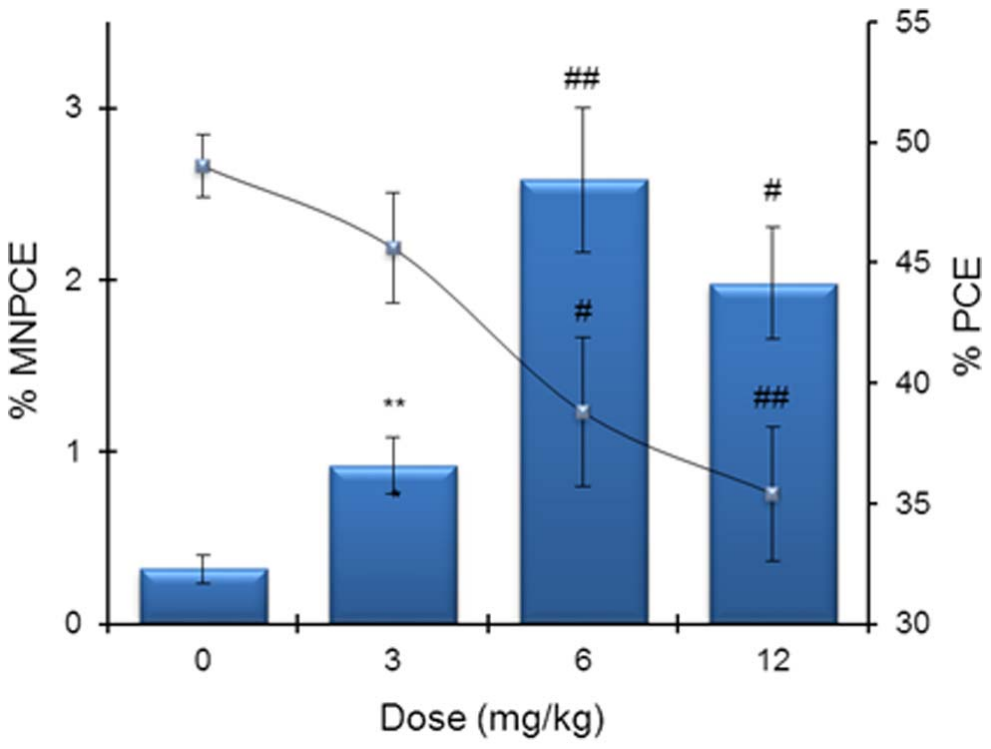
The dose-response effect of epirubicin exposure obtained in the conventional micronucleus test. PCE  =  polychromatic erythrocytes, MNPCE  =  micronucleated polychromatic erythrocytes, shown as blue bars (mean percent ± SD). Reduction of %PCE [(PCE/NCE+PCE) × 100] is shown as solid line (mean percent ± SD). ^#^ *P*<0.05 and ^##^ *P*<0.01 compared with the control group (Kruskal-Wallis test followed by Dunn's multiple comparisons test). **P<0.01 compared with control group (Mann-Whitney *U*-test).

**Figure 2:**
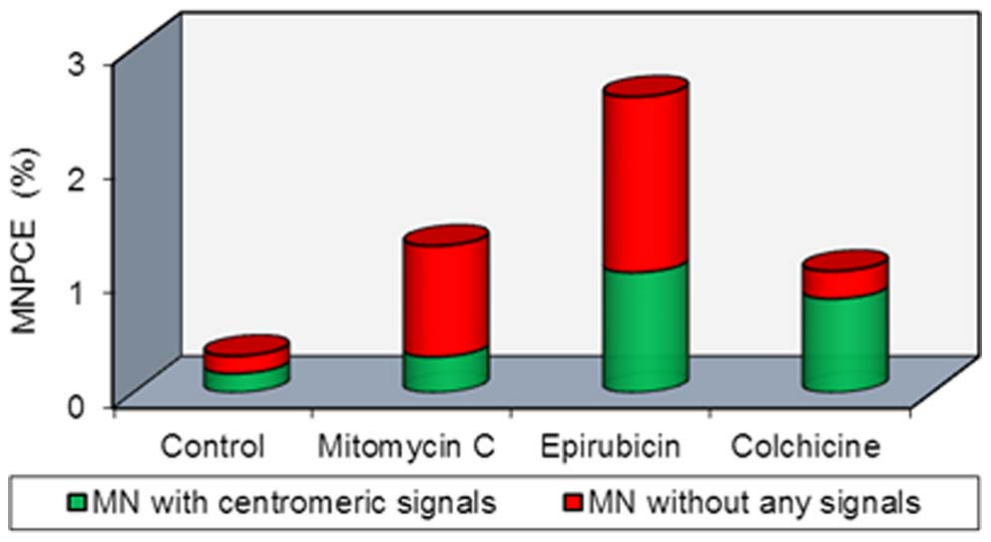
The contribution of clastogenicity (red) and aneugenicity (green) to the induced MN frequencies in animals treated with mitomycin C (2 mg/kg), epirubicin (6 mg/kg) and colchicine (2 mg/kg), MNPCE  =  micronucleated polychromatic erythrocytes.

The incidences of MNPCE in the epirubicin treatment groups and the PCE/NCE ratio are shown in Fig. 1. After treatment with the positive controls colchicine and mitomycin C, a statistically significant increase in the incidence of MNPCE over the control value was observed (Table 1 and Fig. 2). Moreover, the mitomycin C PCE frequencies significantly reduced the percent PCE (Table 1), indicating a reduction in erythroblast proliferation. As seen in Fig. 1, epirubicin at doses of 3, 6 and 12 mg/kg significantly increased the frequency of MNPCE in a dose-dependent manner. The highest frequency of MNPCEs was observed in the 6 mg/kg group. After reaching a peak, there was a decline in the frequency of MNPCEs. At doses of 6 and 12 mg/kg, epirubicin significantly decreased PCE frequencies, indicating a suppression of bone marrow proliferation. On the other hand, epirubicin treatment at a dose of 3 mg/kg did not exhibit any significant differences in the frequency of MNPCE and PCE frequencies compared to the solvent control (P>0.05). In NCEs, the MN frequencies were between 0.02 and 0.05/100 NCE in all groups. Thus, no discrimination between MN induced in PCE and NCE was required for fluorescent analysis of MN with FISH because the NCE only minimally contributed to the total MN number.

**Table 1:** Frequencies of MNPCE in bone marrow of mice 24 h after treatment with the indicated doses of epirubicin, mitomycin C and colchicine.

Treatment groups (mg/kg)	Individual animal scores MN/2000 PCE	% MNPCE (mean ± SD)	% PCE (mean ± SD)
Control	4, 6, 8, 8, 6	0.32±0.08	49.0±2.23
Epirubicin (3)	16, 24, 18, 16, 18	0.92±0.16**	45.6±4.03
Epirubicin (6)	44, 52, 42, 56, 62	2.58±0.42^##^	38.8±4.91^#^
Epirubicin (12)	40, 30, 48, 42, 38	1.98±0.32^#^	35.5±3.15^##^
Mitomycin C (2)	28, 32, 20, 26, 22	1.28±0.23*	43.8±4.14*
Colchicine (2)	18, 16, 24, 32, 16	1.06±0.34*	44.7±5.21

MN  =  micronuclei, PCE  =  polychromatic erythrocytes, MNPCE, micronucleated polychromatic erythrocyte, NCE  =  normochromatic erythrocytes. The mitotic activity was calculated as % PCE  =  [PCE/(PCE + NCE)]×100. ^#^ *P*<0.05 and ^##^ *P*<0.01 compared with the control group (Kruskal-Wallis test followed by Dunn's multiple comparisons test). **P<0.01 compared with control group (Mann-Whitney *U*-test).

### Bone marrow FISH analysis of MN using centromeric DNA-probe

The results of centromeric probe labelling in both PCE and NCE are shown in Fig. 2. In the control group, a total of 67 MN were analyzed, and 35 (52.2%) of them were centromeric-positive. One signal per MN was observed in 51.4%, two signals were observed in 34.3% and ≥3 signals were observed in 14.3%. After the treatment of mice with the positive control aneugen colchicine, 58 (77.3%) of the 75 MN scored were centromere-positive, confirming the predominantly aneugenic effects of colchicine. Of these, 23 MN (39.7%) had one signal, 27 MN (46.5%) contained two signals, and 8 MN (13.8%) had ≥3 signals. After treatment with the positive control clastogen mitomycin C, only 21 MN (24.4%) of 86 MN analyzed were signal-positive, confirming the predominantly clastogenic effects of mitomycin C. Of the signal-positive MN, 12 MN (57.2%) had one signal, 6 MN (28.6%) contained two signals, and 3 MN (14.2%) had ≥3 signals.

In the epirubicin 6 mg/kg group, a total of 128 MN were analyzed by FISH assay and 52 MN (40.6%) were signal-positive, confirming the aneugenic effects of epirubicin. Of these 52 MN, 28 (53.8%) had one signal, 17 (32.7%) contained two signals, and 7 (13.5%) had ≥3 signals. Similarly, epirubicin induced 59.4% signal-negative MN, indicating that they were formed by DNA strand breaks and represented the clastogenic activity of epirubicin. To correlate the FISH data with the conventional MN data, the expected percent of PCE with signals-positive and -negative MN were calculated (Fig. 2). For example, after treatment with MMC, 1.28% of MNPCE were found in the conventional MN test and 24.4% of MN were signal-positive in the FISH analysis, thus 0.31% of the 1.28% MNPCE was calculated to be signal-positive and, correspondingly, 0.96% MN were calculated to be signal-negative. Because the centromere probe labelled mitotic chromosomes at both chromatids, it can be concluded that equal proportions of MN contained single chromatids (chromosome loss) and whole G_2_ chromosomes (non-disjunction).

#### Sperm BrdU-incorporation assay

**Figure 3:**
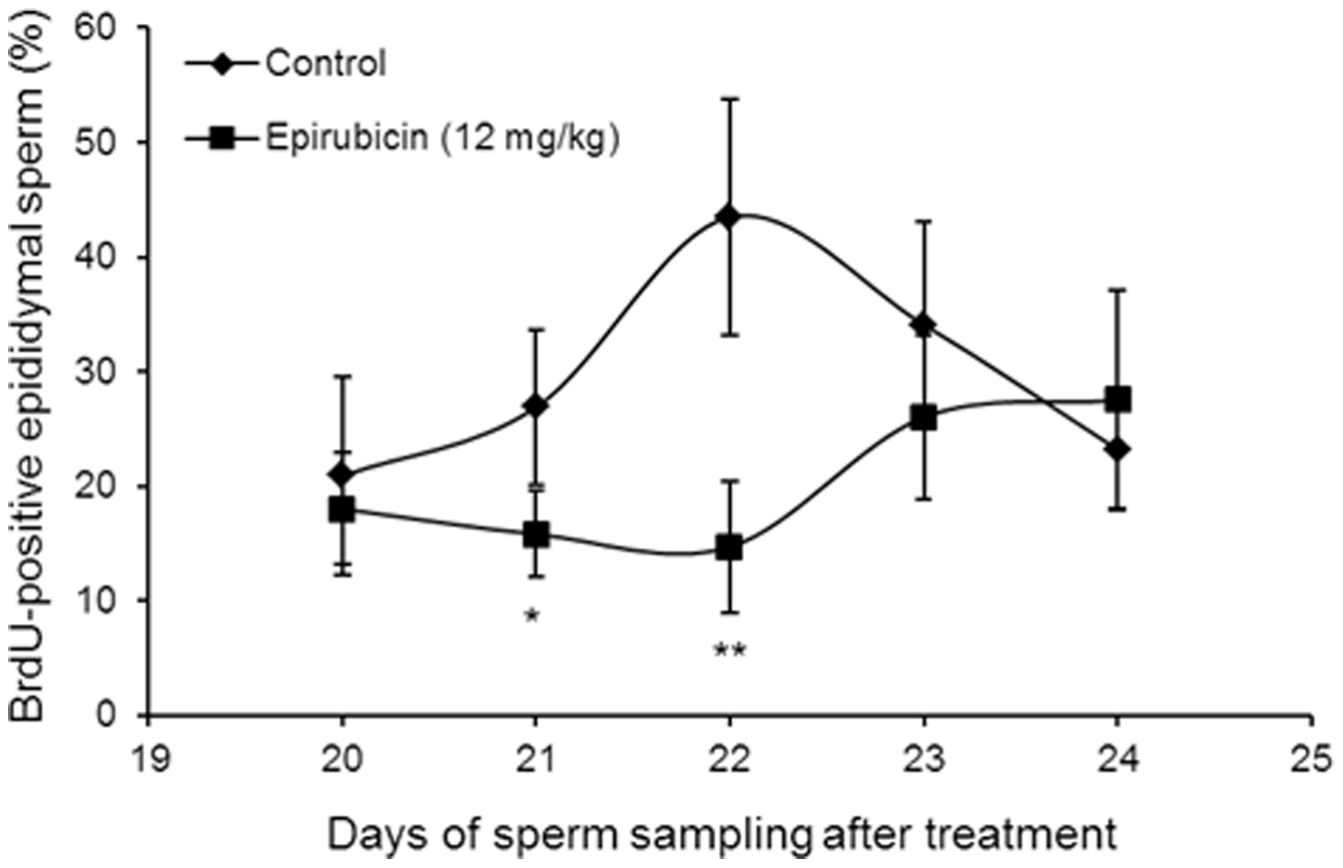
Time course of appearance of BrdU-labelled sperm in the epididymes after treatment with epirubicin. *P<0.05 compared with the concurrent control group (Mann-Whitney *U*-test).

The results of the BrdU-incorporation assay are presented in Fig. 3. With 12 mg/kg of epirubicin, prolongations of the duration of the meiotic divisions were observed. On days 21 and 22, the frequencies of BrdU-labelled sperm in the epirubicin group were significantly below the control values, while on days 23 and 24, there were no significant differences between the epirubicin-treated and control animals. According to Oakberg [23] the development from meiotic spermatocytes of mice to epididymal sperm takes 22 days. Thus, the meiotic delay caused by epirubicin was approximately 48 h. Therefore, the optimum day for sperm sampling in the sperm-FISH assays was concluded to be day 23 with epirubicin.

#### Multicolor sperm FISH assay

**Figure 4:**
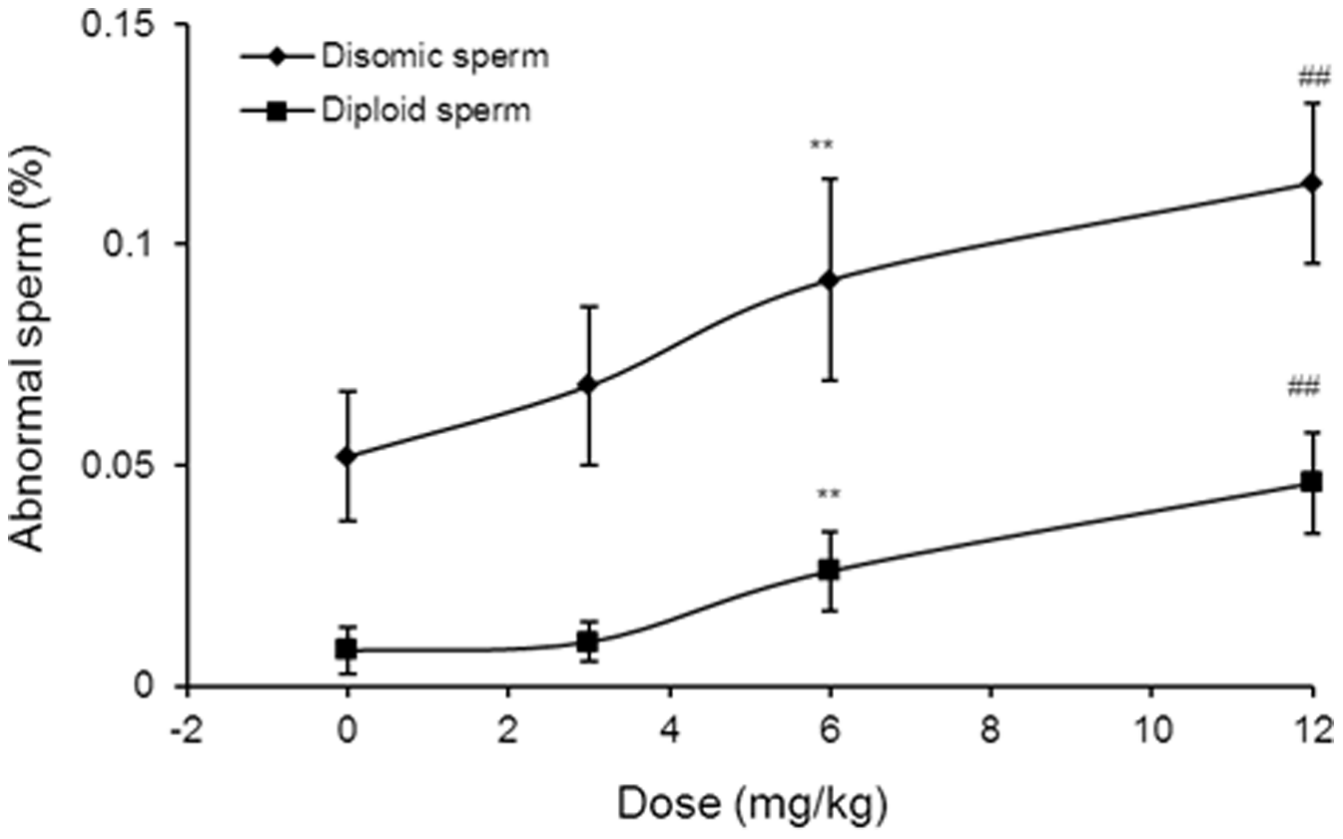
Dose-response curves of the frequencies of abnormal sperm of mice after treatment with epirubicin. ^##^ *P*<0.01 compared with the control group (Kruskal-Wallis test followed by Dunn's multiple comparisons test). **P<0.01 compared with control (Mann-Whitney *U*-test).

**Figure 5:**
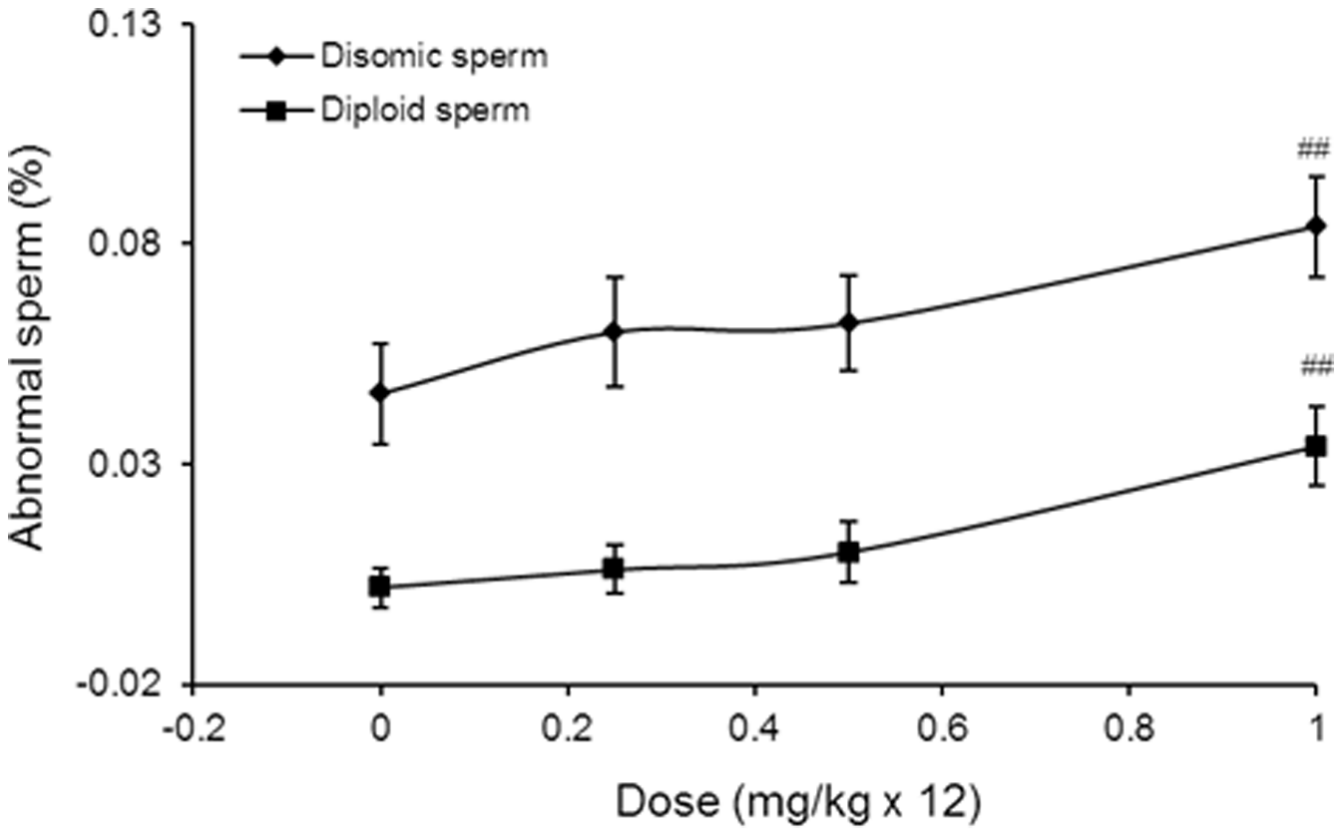
Dose-response curves of the frequencies of abnormal sperm from mice 23 days after the last treatment of epirubicin (daily exposure for 12 consecutive days). ^##^ *P*<0.01 compared with the control group (Kruskal-Wallis test followed by Dunn's multiple comparisons test).

The results of the analysis of aneugenic effects in germ cells of male mice after a single exposure to colchicine and epirubicin are presented in Fig. 4, Table 2 and Table S1. The sex ratios were found to be in the same range as the theoretical ratio of 1∶1 for X- versus Y-bearing sperm in all groups. Significant increases in the frequencies of disomic and diploid sperm were caused by treatment with 6 or 12 mg/kg of epirubicin compared with the corresponding control values. In contrast, the treatment of mice with 3 mg/kg of epirubicin induced no significant increases in disomic or diploid sperm. The frequency of disomic sperm induced by colchicine was significantly increased by a factor of 1.45 compared with the control value. However, in contrast to epirubicin, colchicine did not significantly increase the frequency of diploid sperm, indicating that no complete meiotic arrest occurred (0.006 versus 0.004). The results of multiple exposures to epirubicin are shown in Fig. 5, Table 3 and Table S2. Treatment of mice with 0.25 and 0.5 mg/kg of epirubicin on 12 consecutive days did not induce significant increases of disomic or diploid sperm. However, the highest dose group (1 mg/kg/day), which received a total of 12 mg/kg, induced significant increases in disomic and diploid sperm compared with the corresponding control values.

**Table 2:** Result of the multicolor sperm FISH assay with epididymal sperm of mice treated with colchicine and epirubicin.

	Control	Colchicine (3 mg/kg)	Epirubicin (3 mg/kg)	Epirubicin (6 mg/kg)	Epirubicin (12 mg/kg)
No. of animals	5	5	5	5	5
Sperm scored	50072	50171	50029	50081	50011
X/Y bearing sperm	0.9912	0.9952	1.0011	1.0515	0.9984
Disomic sperm					
X-X-8	8	10	11	14	19
Y-Y-8	6	9	4	10	9
X-Y-8	4	6	5	6	8
X-8-8	2	8	6	10	13
Y-8-8	6	5	8	6	8
Total	26	38	34	46	57
% Disomies ± SD Diploid sperm	0.052±0.014	0.076*±0.005	0.068±0.017	0.092**±0.022	0.114##±0.018
X-Y-8-8	0	1	1	2	5
X-X-8-8	1	2	2	6	13
Y-Y-8-8	1	0	1	5	5
Total	2	3	4	13	23
% Diploidies ± SD	0.004±0.005	0.006±0.005	0.008±0.004	0.026**±0.008	0.046##±0.011

## *P*<0.01 compared with the control group (Kruskal-Wallis test followed by Dunn's multiple comparisons test). **P<0.01 compared with control (Mann-Whitney *U*-test).

**Table 3:** Result of the multicolour FISH assay with epididymal sperm of mice treated with epirubicin (Daily exposure for 12 consecutive days).

	Control	Epirubicin (0.25 mg/kg)	Epirubicin (0.5 mg/kg)	Epirubicin (1 mg/kg)
No. of animals	5	5	5	5
Sperm scored	50012	50078	50012	50171
X/Y bearing sperm	1.0049	1.0044	1.0049	0.9952
Disomic sperm				
X-X-8	7	9	7	11
Y-Y-8	5	6	6	10
X-Y-8	2	3	3	5
X-8-8	6	6	7	8
Y-8-8	3	6	8	8
Total	23	30	31	42
% Disomies ± SD Diploid sperm	0.046±0.011	0.06±0.012	0.062±0.01	0.084##±0.011
X-Y-8-8	0	1	1	4
X-X-8-8	0	1	2	6
Y-Y-8-8	1	1	2	7
Total	1	3	5	17
% Diploidies ± SD	0.002±0.004	0.006±0.005	0.010±0.007	0.034##±0.008

## *P*<0.01 compared with the control group (Kruskal-Wallis test followed by Dunn's multiple comparisons test).

## Discussion

The majority of chemotherapeutic drugs are specifically designed to interfere with DNA synthesis, cellular metabolism, and cell division. Due to this mode of action, these drugs are expected to cause several types of mutations [24]. Mutagenicity of chemotherapeutic drugs to normal cells is one of the most serious problems in chemotherapy due to the possibility of inducing secondary malignancies and abnormal reproductive outcomes such as Down, Klinefelter and Turner syndromes. Therefore, it is imperative to determine the mutagenic effect of chemotherapeutic drugs on normal cells. The micronucleus test has been widely used to measure unrepaired genome damage, and increased MN frequency predicts the risk of cancer in humans [25]. As MN can result from chromosome breakage (clastogenicity) or lagging chromosomes (aneugenicity), the detection of MN has the potential to be used as a screen for numerical chromosomal aberration induction if assays that allow the identification of entire chromosomes inside of MN, such as the FISH assay, are included.

The clastogenic activity of epirubicin during mitosis *in vivo* was reported by the bone marrow MN test [15]. At present, the origin of the epirubicin-induced MN has not yet been determined. Therefore, the current study was designed to investigate the ability of epirubicin to induce aneuploidy in somatic and germinal cells of male mice. To determine the efficiency of the method, two model mutagens, colchicine and mitomycin C, known to have aneugenic and clastogenic actions, respectively, were used as positive controls. The observed results of the MNPCE and the distribution of signals per MN in the control and the two positive mutagens in the current study correspond well with the published data [16], [18]. These data confirmed the sensitivity of the experimental protocol followed in the detection of genotoxic effects.

The results of the MN test have shown that epirubicin produces dose-dependent increases in MN formation in mouse bone marrow *in vivo* (Fig. 1) and an increase in centromeric-negative and centromeric-positive stained MN, indicating the induction of both clastogenicity and aneugenicity (Fig. 2). Both the clastogenic and the aneugenic potential of epirubicin in somatic cells can give rise to the development of secondary tumors. The results of clastogenicity confirm the findings of previous *in vivo* studies, where increases in MN formation and structural chromosomal aberrations in mouse somatic cells over a similar dose range were observed after epirubicin treatment [15]. The results of these studies also agree with the previously reported micronucleus induction by epirubicin in murine cancer cells *in vitro* [26], in the erythrocytes of incubated hen's eggs [27], and in the human lymphoblastoid TK6 cell line [28]. Moreover, the induction of structural and numerical chromosomal aberrations was also reported previously in cancer patients receiving epirubicin-containing chemotherapy [29]. Previous studies have also shown that epirubicin induces structural chromosomal aberrations in cultured HeLa cells [30], both structural and numerical chromosomal aberrations and sister chromatid exchanges in a Chinese hamster cell line [13], and chromosomal aberrations in peripheral blood lymphocyte cultures from women with breast cancer treated by a epirubicin-containing regimen *in vitro* [31].

Studies in humans have shown that certain chemotherapy regimens increase the frequency of aneuploidy in germinal cells [8], [32], suggesting that such patients may be at higher risk for abnormal reproductive outcomes, particularly in the reproductive ages. Therefore, it is of general concern to decrease the risk of aneuploidy production, detect germ cell aneugens and understand the causal mechanisms. In the current study, aneuploidy was determined in germinal cells by the sperm-FISH assay with DNA probes specific for mouse chromosomes 8, X and Y, each labeled with a different color. To determine the reliability of the methods, colchicine, known to be predominantly aneugenic, was used as positive control substance, and the results of the positive and negative control were in the same range as those of earlier studies [21], [33]–[35]. These data confirmed the sensitivity of the experimental protocol in the detection of aneuploidogenic effects of the tested compounds.

Epirubicin is most active in the S and G_2_ phases of the cell cycle; however, it has some detectable activity in all phases of the cell cycle [36]. It has often been reported that chemicals with aneugenic properties can alter the progression of cell division in both meiotic and mitotic cells [37]. In the current study, the time of development from meiotic divisions in spermatocytes to epididymal sperm was assessed by the BrdU-incorporation assay. The results clearly indicate that epirubicin prolonged the duration of meiotic divisions in mouse spermatocytes for 48 h (Fig. 3). These observations therefore confirm previous findings that epirubicin-induced inhibition of topoisomerase II function during different phases of the cell cycle slows down cell cycle progression and causes cells to arrest at the G_2_/M phase [36]. Such G_2_/M arrest may be due to induction of G_2_ checkpoint machinery, which allows damaged DNA to be repaired before cells move to the next cell cycle stage [38].

The *in vivo* information on the effects of epirubicin on non-disjunction during meiosis is limited, but certain other anthracyclines, such as doxorubicin and its derivative idarubicin, prevent chromosomal segregation and induce significant increases in the frequencies of disomic and diploid sperm [35]. Moreover, an *in vitro* study showed that the treatment of Chinese hamster cultures with epirubicin induced numerical aberrations in the form of hypodiploidy and hyperdiploidy [13]. In addition, flow cytometric and histological analysis of mouse spermatogenesis showed an increase of the coefficient of variation in the DNA histogram as a measure of aneuploidy, and an increase of diploid spermatids after treatment with epirubicin [39]. In agreement with the above-cited reports, the present experiment showed that exposure to epirubicin caused significant dose-dependent increases in the frequencies of disomic and diploid sperm and that the induction of aneuploidy was linearly dose-responsive between 0 and 12 mg/kg of epirubicin (Fig. 4). These *in vivo* observations are also in line with an earlier *in vitro* report on human lymphocytes cultured from healthy individuals and cancer patients in whom doxorubicin exposure caused an increase in the trisomies of chromosomes 7 and 17 [40]. Furthermore, Ganapathi et al. [41] reported that human leukemia HL-60 cells that carry monosomy 8 as the only karyotypic change acquired 7q21 markers following exposure to doxorubicin. Additionally, cytogenetic findings showed trisomy 8 in patients who received courses of systemic chemotherapy with anthracycline [42]. Monosomy 7, 7q-, and unbalanced translocation including chromosome 7 were observed in patients who received anthracycline-containing chemotherapy [43]. Moreover, structural chromosomal aberrations of chromosomes 1, 9 and 16 have been related to chemotherapeutic drugs containing anthracycline [44].

Among other classes of topoisomerase II inhibitors, etoposide has been studied previously in mouse spermatocytes [45], [46]. The results of these studies indicate that etoposide acts as a genotoxic agent and induces aneuploidies in primarily meiotic germ cells after treatment of pachytene cells. On the other hand, Kallio and Lähdetie, [47] found that sensitivity to etoposide in mice was greatest during diplotene-diakinesis of primary spermatocytes, reduced during late pachytene and low during preleptotene stages; a very different pattern than with DNA alkylating chemicals. These authors suggested that etoposide caused a failure of resolution of recombined chromosome arms, probably associated with cell cycle arrest and triggering of the apoptotic pathway. In their detailed cytogenetic studies, Marchetti et al. [45] reported that pachytene was the most sensitive stage of spermatogenesis for the induction of structural chromosome aberrations and aneuploidy. Since the data of Schmid et al. [22] and those of Kallio and Lähdetie [47] could demonstrate that etoposide prolonged the meiotic cell cycle, it seems possible that the effects seen in first cleavage divisions by Marchetti et al. [45] in the 24.5 days mating group may have actually been induced at a later stage, i.e. during meiotic diakinesis, MMI or MMII instead of pachytene as the authors suggested.

In the current study, we planned sperm-FISH study to determine if subacute treatment with low doses of the topoisomerase II inhibitor epirubicin would have an effect because earlier prophase stages would be included in the epirubicin treatment. Individual doses of 0.25, 0.5 and 1 mg/kg of epirubicin were injected on 12 consecutive days, and sperm were sampled 23 days after the last treatment with epirubicin. A total of 12 mg/kg epirubicin applied to the entire prophase of meiosis significantly increased disomic and diploid sperm frequencies, while a total dose of 3 and 6 mg/kg doxorubicin were negative (Fig. 5). In contrast, a single dose of 6 mg/kg epirubicin applied to spermatocytes during MMI/MMII gave a positive result (Fig. 4). These data suggest that earlier prophase stages contribute relatively less to epirubicin-induced aneuploidy in male germ cells.

In the present study, it was found that epirubicin caused noticeable increases of autodiploid sperm (XX88 and YY88). After treatment with epirubicin, autodiploid sperm resulting from arrest of MMII were more frequent than diploid sperm resulting from arrest during MMI (XY88). Thus, the second meiotic division was more sensitive to epirubicin treatment than the first meiotic division. The conclusion that second meiotic divisions were more sensitive to epirubicin treatment than first meiotic divisions is also supported by the observed frequencies of disomic sex chromosomes. Sperm with signals of XX8 or YY8 were more frequent than sperm with signals of XY8. These observations confirm that the sperm-FISH assay for disomy or diploidy is capable of detecting effects induced during both meiotic divisions and to compare the sensitivity of both meiotic divisions, as previously reported [48], [49].

In the present sperm FISH assay, it was found that the lowest positive dose to cause disomic or diploid sperm was 6 mg/kg of epirubicin. However, mouse bone marrow MN studies showed that exposure to 3 mg/kg epirubicin yielded a significant increase in MNPCE. This observation suggests that MN in bone marrow are induced at lower doses than disomies or diploidies in sperm. Hence, the bone marrow is the more sensitive tissue. However, the assays measure different end-points. Chromosome loss and breakage is measured in the MN test, and non-disjunction is detected in the sperm-FISH assay. Therefore, the present data confirm the general paradigm of hazard assessment, which states that a positive outcome of the bone marrow MN test is an indicator of the genotoxic potential of a compound in germ cells. However, quantification of aneuploidy in germ cells is important for risk assessment purposes.

DNA topoisomerase inhibitors have a clear tendency to cause double-stranded DNA breaks, which primarily result in the formation of centromere-negative MN [16]. Similarly, the demonstration that epirubicin is an effective topoisomerase II inhibitor suggests that epirubicin elicits its clastogenic effects through this mechanism. Epirubicin metabolites may also activate cells to enhance intracellular production of reactive oxygen species, of which the stable and diffusible forms can damage nuclear DNA [50]. If the cellular repair mechanisms are overloaded, primary DNA damage may lead to structural or numerical chromosomal aberration and eventually cause tumors [51]. This suggestion is supported by the fact, that individuals who developed a second malignant neoplasm after treatment for a first malignant neoplasm had a lower ability to repair DNA double-strand breaks than those that did not develop a second malignant neoplasm as measured by γH2AX intensity [52]. The induction of centromere-positive MN by epirubicin indicates that there may be another mechanism through which epirubicin induces its genotoxic effect. This observation underscores the importance of using the FISH modification of the MN assay to determine the origin of the induced MN. The potential mechanism by which epirubicin may exert its aneugenic effect is through inhibition of topoisomerase II, which can result in missegregation of chromosomes during cell division. This occurs because DNA topoisomerase II is necessary for the proper separation of sister chromatids during cell divisions with both non-disjunction and breakage occurring in its absence [53], [54]. Thus, epirubicin may be able to inhibit two key roles of topoisomerase II; its ability to properly segregate newly replicated chromosomes as well as its function in re-ligating transient double-stranded DNA breaks.

In summary, by using the FISH assay with a centromeric DNA probe for erythrocyte MN, it was shown that epirubicin is not only clastogenic but also aneugenic in somatic cells *in vivo*. By using the BrdU-incorporation assay, it was shown that the meiotic delay caused by epirubicin was approximately 48 h. With the sperm-FISH analysis, it was shown that epirubicin induces aneuploidies during meiosis that result in disomic sperm as well as complete meiotic arrest that creates diploid sperm. The prevalence of autodiploid (XX88, YY88) and disomic (XX8 or YY8) sperm indicates that the second meiotic division is more sensitive to epirubicin than the first meiotic division. The results also suggest that earlier prophase stages contribute relatively less to epirubicin-induced aneuploidy. Both the clastogenic and aneugenic potentials of epirubicin can give rise to the development of secondary tumors and abnormal reproductive outcomes in cancer patients and medical personnel exposing to drug regimens that include epirubicin. Although epirubicin has less systemic and cardiac toxicity than doxorubicin and other anthracyclines with an equivalent spectrum of antitumor action [55], it does have aneuploidogenic and cytotoxic effects in non-tumour cells. This aneuploidogenic effect of the drug may be responsible for the appearance of abnormal reproductive outcomes and secondary tumors, which are noticed in some cancer patients sometime after successful treatment of their primary cancers with epirubicin-containing chemotherapy.

## Supporting Information

Table S1 **Results of the multicolor sperm FISH assay with epididymal sperm of mice treated with acute doses of colchicine and epirubicin (Individual animal scores).**
(XLS)

Table S2 **Results of the multicolor sperm FISH assay with epididymal sperm of mice treated with subacute doses of epirubicin (Individual animal scores).**
(XLS)
