# Correction: Aneugenic Effects of Epirubicin in Somatic and Germinal Cells of Male Mice

**DOI:** 10.1371/journal.pone.0118048

**Published:** 2015-02-19

**Authors:** The PLOS ONE Staff

There are errors in the author affiliations. The affiliations should appear as shown here: Sabry Mohamed Attia^1,3^*, Sheikh Fayaz Ahmad^1^, Radwa Mohamed Okash^2^, Saleh Abdulrahman Bakheet^1^

1 Department of Pharmacology and Toxicology, College of Pharmacy, King Saud University, Riyadh, Saudi Arabia, 2 Laboratory of Chemical and Clinical Pathology, Ministry of Health, Nasr City, Cairo, Egypt, 3 Department of Pharmacology and Toxicology, College of Pharmacy, Al-Azhar University, Cairo, Egypt.
